# Analysis of magnetic structures in *JANA2020*

**DOI:** 10.1107/S2052520624008163

**Published:** 2024-09-19

**Authors:** M. S. Henriques, V. Petříček, S. Goswami, M. Dušek

**Affiliations:** a Institute of Physics of the Czech Academy of Sciences Na Slovance 2 182 21Prague Czechia; Oak Ridge National Laboratory, USA

**Keywords:** magnetism, superspace, modulations, crystallography

## Abstract

*JANA2020* is a program developed for the solution and refinement of regular, twinned, modulated, and composite crystal structures. In addition, *JANA2020* also includes a magnetic option for solving magnetic structures from powder and single-crystal neutron diffraction data. This tool uses magnetic space and superspace symmetry to describe commensurate and incommensurate magnetic structures. The basics of the underlying formulation of magnetic structure factors and the use of magnetic symmetry for handling modulated and non-modulated magnetic structures are presented here, together with the general features of the magnetic tool. Examples of structures solved in the magnetic option of *JANA2020* are given to illustrate the operation and capabilities of the program.

## Abstract 2

The magnetic option of the crystallographic program *JANA2020* is a specific tool for solution, analysis, and description of modulated and non-modulated magnetic structures.

## 1. Introduction

The crystallographic computing system *JANA2020* is a program created for structure analysis of polycrystalline or single-crystal materials from diffraction data (Petříček *et al.*, 2023 ▸). *JANA* has been developed over the last 40 years and is a well established tool in the crystallographic community. Its history, general features, structure, and latest improvements can be found in Petříček *et al.* (2014 ▸, 2023 ▸).

*JANA2020* allows the solution and refinement of crystal and magnetic structures, standard or modulated (up to three modulation vectors), commensurate and incommensurate, twinned or composite. It is also possible to simultaneously refine the crystal and the magnetic structures, combine various data types, and use them for the same structure model.

The magnetic option dedicated to the determination and description of magnetic structures is one of the swiftly developing implementations in *JANA2020*. This tool is continuously improved and tested with experimental data and feedback from neutron users and instrument experts worldwide. The formalism and methodology behind this specialized routine in *JANA* support a comprehensive characterization and description of magnetic structures found in different magnetic materials.

‘Magnetic material’ is a broad term that generally refers to the existence of magnetic order in a material, that is, the onset of an orderly long-range arrangement of magnetic moments below a certain critical temperature at which the magnetic exchange interaction holds over (or pairs up with) other energy terms. Magnetic phases and transitions are important and current topics in different research fields, from fundamental to applied physics or materials science (Guillou *et al.*, 2018 ▸).

The ordering of magnetic moments in a crystalline material is intrinsically linked to the underlying symmetry of the crystal lattice that characterizes the position periodicity of the atoms carrying magnetic moments within the material. Generally, the crystal structure (also termed nuclear structure) of the paramagnetic phase is known *a priori* and often termed parent structure. The information on the translation properties of the moment distribution in a crystal is described by vector quantities, the so-called magnetic propagation vectors **k***_i_*. This wavevector expresses the relation between the spin^1^ of a general atom ν in the unit cell *n* and the spin of the symmetry equivalent atom ν in the *zero*th cell. It is a reciprocal lattice vector within or on the surface of the first Brillouin zone of the Bravais lattice of the parent (paramagnetic) unit cell. This concept provides a simple and concise formalism for the description of magnetic structures according to the modulus and number of propagation vectors needed to represent the magnetic ordering. The magnetic structure is said to be non-modulated if **k** = 0. That is, the periodicity of the magnetic structure coincides with the periodicity of the paramagnetic crystal structure. If the magnetic arrangement has a non-zero propagation vector, the structure is said to be modulated. In this case, the magnetic unit cell can be commensurate (**k** is rational) or incommensurate (**k** is irrational) with the underlying crystalline (parent) lattice. An exclusive position among commensurate magnetic structures is held by those whose vector **k** is not a vector of a primitive reciprocal cell, but the vector 2**k** is. These lead to full compensation of magnetic moments in antiferromagnetic arrangement, and their symmetry is included in magnetic (Shubnikov) space groups. In the following, we will limit the commensurate magnetic structure to these cases only. General commensurate modulation vectors can be fully described as magnetic commensurately modulated with magnetic space groups. Furthermore, magnetic modulated phases can be single-*k* or multi-*k* structures, whether only one or more independent propagation vectors are needed to describe the spin arrangement. Most of the reported magnetic structures are single-*k*. For this reason and for the sake of simplicity, the discussion presented here is restricted to one-dimensional modulations.

In the framework of the Landau (1937 ▸) theory, a magnetic phase transition involves a symmetry reduction from the paramagnetic (parent) phase to the magnetically ordered one. A symmetry-based description of a magnetic phase includes the assignment of the relevant symmetry modes for the spin configuration and their constraints consistent with the parent phase and the magnetic propagation vector(s). In this context, the two approaches most widely used to describe magnetic structures are group-representation analysis and magnetic symmetry. Group-representational analysis is a method in which the set of possible magnetic configurations is given by the spin modes transforming as one or more physically irreducible representations (irreps) of the paramagnetic space group (Bertaut, 1968 ▸). In the second approach, the magnetic symmetry of a particular phase can be expressed in the form of a magnetic (Shubnikov) space group (MSG) (Koptsik, 1966 ▸; Bradley & Cracknell, 1972 ▸) for commensurate ordering or magnetic superspace groups (MSSG) for incommensurate order (Janner & Janssen, 1980 ▸; Petříček *et al.*, 2010 ▸; Perez-Mato *et al.*, 2012 ▸). The symmetry group of a magnetic phase comprises all the symmetry restrictions for the spins and other important features such as magneto-structural couplings, formation of domains and twin-related configurations.

In the magnetic option of *JANA2020*, the symmetry information from MSGs and MSSGs is applied to derive the magnetic structure factors, analyze the symmetry of the diffraction data, and constrain magnetic and crystal parameters. The user can explore and test the different magnetic configurations against the experimental data. The selection and analysis of the different models can be done through the simulation of powder profiles, direct visualization, or by calling in external programs. The magnetic option of *JANA2020* is the only existing tool capable of handling modulated magnetic structures coupled with secondary (structural) modulations.

This paper presents details on how *JANA2020* works with magnetic structures. We explain the magnetic structure factors formulated in *JANA2020* and the conditions for magnetic diffraction from non-modulated and generally modulated phases, followed by the symmetry of magnetic diffraction and a brief description of the magnetic space and superspace groups. Another section is devoted to representation analysis as implemented in *JANA2020*. The tools and capabilities of the magnetic option of *JANA2020* are detailed for powders and single crystals using illustrative examples.

## 2. Magnetic structure factor in *JANA2020*

Let us consider a general long-range ordering of magnetic moments carried by some atom(s) in a three-dimensional regular crystal. The magnetic scattering from such a periodic array of atoms gives information about the moment distribution in the material. Independently of the type of ordering, the atomic magnetization density ρ_mag_(**r**) is a periodic vector function of space. For the case that crystal and magnetic structures have the same periodicity, that is **k** = 0, the magnetization density can be Fourier expanded according to 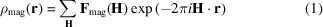
where the Fourier coefficients **F**_mag_(**H**) are the magnetic structure factors and **r** is a positional vector. The summation runs over all the diffraction vectors **H** = 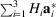 in a three-dimensional lattice defined by the reciprocal basis vectors 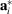. Considering the contribution ρ_ν, mag_ (**r**) of each magnetic atom ν located at **r**_ν_ in the unit cell *n* of the structure to the magnetization density, then
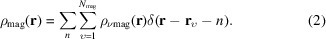


The first sum runs over the unit cells in the crystal, *n* = 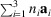, whereas the second summation is taken over the *N*_mag_ magnetic atoms in the reference unit cell. Therefore, the magnetic structure factor **F**_mag_(**H**) is
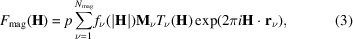
where *f*_ν_(|**H**|) and *T*_ν_(**H**) are the magnetic form factor and the anisotropic displacement parameters of the magnetic atoms with moment **M**_ν_ in the unit cell, respectively. The constant *p* is equal to 0.2696 × 10^−12^ cm and unifies the scales by converting the units of the magnetic structure factor (Bohr magnetons) to the neutron scattering cross-section (barns).

Magnetic structures characterized by **k** ≠ 0 and at least one irrational component do not possess translation periodicity in real space. However, the periodical perturbations generally incommensurate with the underlying three-dimensional lattice can be conveniently described by the superspace formalism (de Wolff *et al.*, 1981 ▸). That is, the magnetization density of an incommensurable modulated magnetic structure is embedded into a higher-dimensional space (superspace or internal space) to recover the translational symmetry. This allows the introduction of a general structure factor by an equation similar to (1).

For any single-*k* modulated structure, the magnetic moment of the atom ν can be expressed as a Fourier series in the form
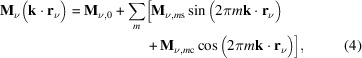
where **M**_ν__,0_ is the absolute term, **M**_ν__,*m*s_, and **M**_ν__,*m*c_ are the amplitudes of the sine and cosine terms, respectively. The structure factors derived from the kinematic theory of diffraction account for sharp diffraction spots located at reciprocal points **H** and **Q** = **H** ± *m***k** (*m* > 0):
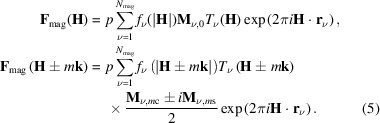


These formulae are valid for the most frequent case of a magnetic modulation not coupled with any secondary atomic modulation. Then, the magnetic structure factors can be derived analytically, and each *n*-th harmonic in the Fourier expansion (4) leads to satellite diffraction of the *n*-th order. Magnetic and positional and/or occupational modulations can also be treated, but it requires the use of an integration method over the internal space to model their combined effects properly (Yamamoto, 1982 ▸). These complex cases are rare, but proper magnetic and structural incommensurability in an organometallic phase was recently solved in *JANA2020* (Cañadillas-Delgado *et al.*, 2020 ▸).

One of the most effective techniques to probe magnetic ordering at the atomic level is neutron diffraction. Neutrons interact with the nuclei, yielding information on the nuclear (crystal) structure, and also interact with the unpaired electrons on the outer shells of the magnetic atoms, providing details about the magnetic structure. Notably, these two contributions are independent if non-polarized neutron beams are used. Thus, the overall observed intensity in a diffraction experiment is just the sum of the nuclear and magnetic components:
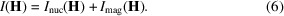


The intensity of magnetic diffraction is related to the magnetic structure factor by the fundamental formula of Halpern & Johnson (1939 ▸)
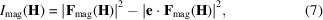
where **e** = **H**/|**H**| is the unit vector along the scattering vector **H**. That is, magnetic diffraction intensity is not directly proportional to the absolute value of the structure factor but depends on the direction of the magnetic moments with respect to the incident neutron beam. Magnetic intensity is observable only for the moment components that are not parallel to the scattering vector.

Expression (6) enables the use of the diffraction data to solve magnetic structures in two different ways. Some programs apply a multiphase approach by treating nuclear and magnetic phases separately. On other tools, the nuclear and magnetic contributions are combined in parallel during the refinements and used to produce a complete model. *JANA2020* uses the latter method.

## 3. Symmetry description of magnetic structures: magnetic space and superspace groups

Magnetic structures can be fully described by means of magnetic space (or superspace) groups akin to the space groups used in conventional crystallography. In fact, a magnetic space (Shubnikov) group describing a commensurate magnetic ordering contains the crystal symmetry elements combined with a time inversion operator (Belov *et al.*, 1957*a* ▸,*b* ▸). Any magnetic symmetry operation **Ŝ** can be written as
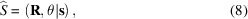
where **R**, θ = ±1, and **s** are the rotation matrix (proper or improper), the time inversion operator (also called time reversal or spin reversal), and the translation part of **Ŝ**, respectively. The symmetry elements combined with the time inversion are called primed to indicate that the inversion of the spin sign is to be included in the conventional action of the operator. A paramagnetic space group is the direct product of the nuclear space group and time reversal represented symbolically by 1′. These groups describing the disordered spin state are termed gray groups. The symmetry groups allowing some spin orderings are the *proper magnetic groups* (Souvignier, 2006 ▸), and they do not include the pure time inversion operation **Ŝ** = (**E**, −1|0). The magnetic moments of two atoms μ and ν with positions related by **r**_μ_ = **Ŝr**_ν_ = **Rr**_ν_ + **s** transform (as axial vectors) under **R** according to
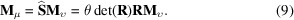


The 1651 Shubnikov space groups are obtained by combining time reversal with the 230 crystallographic space groups (Koptsik, 1966 ▸; Bradley & Cracknell, 1972 ▸). The MSGs are grouped into four types according to whether time reversal is included and, if present, whether it acts over lattice rotations or translations. First, the type I groups do not contain the time inversion operation and correspond to the conventional crystallographic groups. The type II groups are the gray groups with all the operators primed and unprimed. The groups where all rotations appear with and without time inversion are of type III. Last, the type IV groups have half of the translations combined with time reversal.

Two notations are yet most currently used for the Shubnikov groups, corresponding to two different derivations of the magnetic unit cell: the Belov–Neronov–Smirnova (BNS) (Belov *et al.*, 1957*b* ▸) and the Opechowsky–Guccione (OG) (Opechowsky & Guccione, 1965 ▸). The BNS setting describes the symmetry operations in a unit cell (supercell), defining the lattice periodicity of the spin arrangement, which is generally different from the paramagnetic one. The OG setting uses a unit cell of the parent phase that does not reproduce the lattice of the magnetic configuration. These two notations coincide for the groups of type I–III and affect only type IV. Both notations carry different robust and weak aspects recently addressed and combined in a new unified notation for MSGs: the UNI symbols (Campbell *et al.*, 2022 ▸). The magnetic tools of the Bilbao Crystallographic Server (https://www.cryst.ehu.es) list MSGs (and magnetic point groups) in the BNS, OG, and UNI settings. Most computational tools for magnetic structure determination, including *JANA2020*, still use mostly the BNS notation based on the computer-readable tables available at ISO-MAG (Stokes & Campbell, 2010 ▸). Nevertheless, there are plans to include the UNI symbols in *JANA2020* soon.

As magnetic phase transitions involve symmetry-breaking, some symmetry operations of the parent space group do not belong to the space group of the ordered phase. Instead, the lost symmetry elements relate to the magnetic domains created in response to the symmetry lowering.

The concept of magnetic space group presented above has been generalized to modulated magnetic phases (Janner & Janssen, 1980 ▸) by employing the superspace formalism in a way similar to modulated crystal structures (Perez-Mato *et al.*, 2012 ▸; Petříček *et al.*, 2010 ▸). Within this approach, any modulated magnetic structure with a single propagation **k** is fully characterized by a basic periodic lattice with symmetry given by a Shubnikov group, plus the set of modulation functions periodic in the superspace for the magnetic moments of the atoms in the basic unit cell. In terms of symmetry, this translates to a superspace operation between modulation functions:
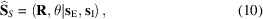
where the rotation matrix has the dimension (3+1)×(3+1) and the vector **s** (3+1). The matrix form shows that the superspace groups are a 3×1 reducible subset of the general four-dimensional space groups (de Wolff *et al.*, 1981 ▸):
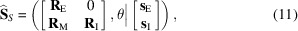
where **R**_E_, **R**_M_, **R**_I_ are external 3×3, mixed 1×3, and internal 1×1 block matrices, respectively; **s**_E_ and **s**_I_ are external 3×1 and internal 1×1 blocks of the translation part. The external parts are determined from the basic symmetry of the main reflections in the diffraction pattern. The non-zero rotational matrices are related by the propagation vector of the structure:
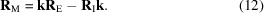


The lattice translation in the real (external) three-dimensional space is **s**_E_, and such that (**R**_E_, θ | **s**_E_) is an ordinary symmetry element of the paramagnetic space group as defined in (8).

The set of symmetry elements imposes constraints on the form of the modulation functions and relates them for the symmetry-equivalent atoms in the basic structure. The spin modulation function of two atoms μ and ν symmetry related as **r**_μ_ = **R**_E_**r**_ν_ + **s**_E_ is given by
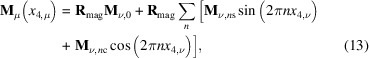
where
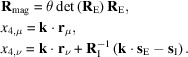


It is derived from these expressions that in the case of μ = ν, the possible modulation functions are forced to have specific orientations.

**Figure 1:**
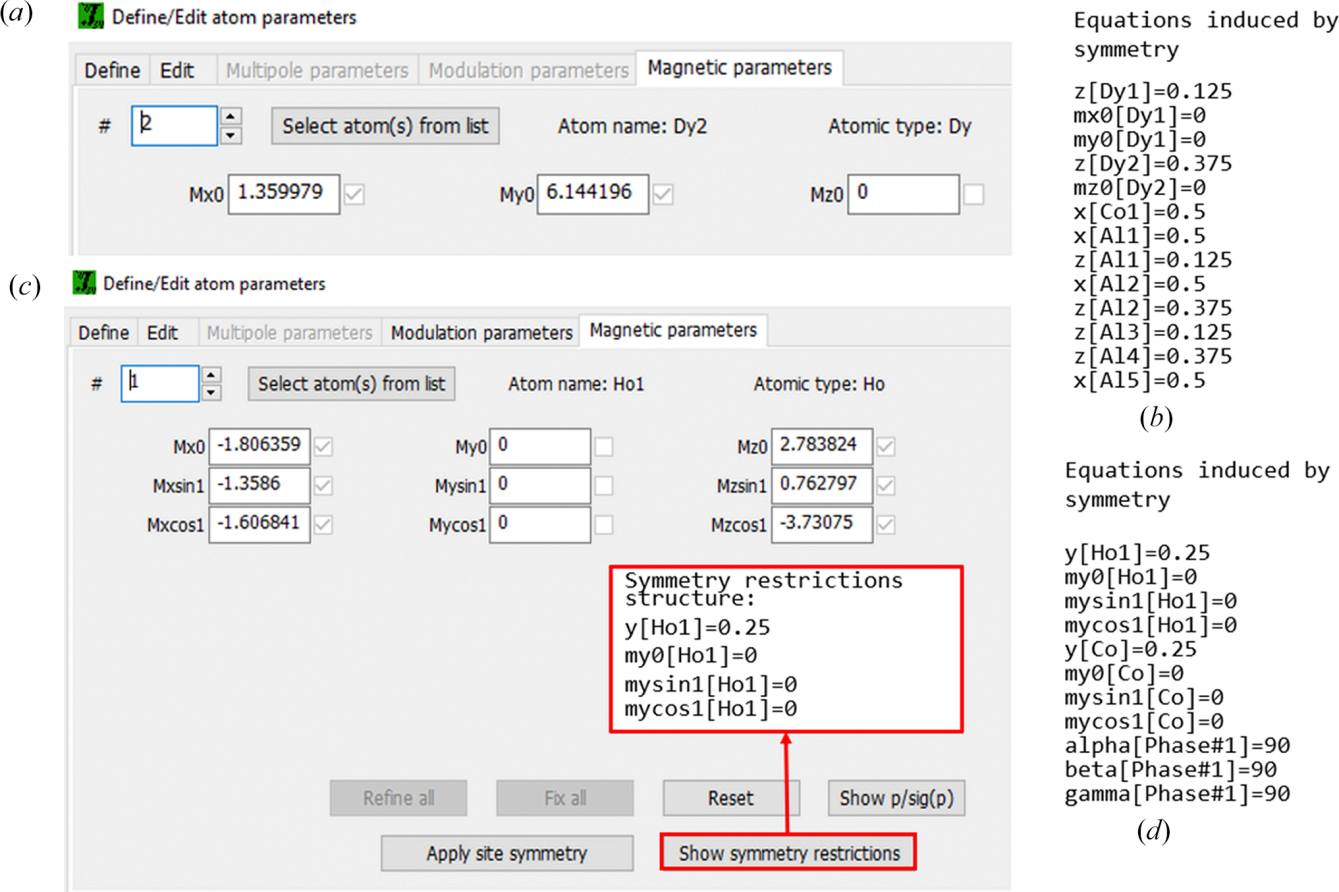
Symmetry restrictions as presented in the magnetic option of *JANA2020* for the magnetic structures of (*a*,*b*) Dy_2_Co_3_Al_9_ (commensurate, 2 K) (Gorbunov *et al.*, 2018 ▸) and (*c*,*d*) Ho_3_Co (incommensurate, 18 K) (Goswami *et al.*, 2024 ▸). The listing of restrictions used during refinements can be consulted for each magnetic atom through the button ‘Show symmetry restrictions’ (*a*,*c*); or for all magnetic (and non-magnetic) atoms in the unit cell in the refinement listing (*b*,*d*).

In the magnetic option of *JANA2020*, all the symmetry restrictions for any structure, modulated or not, are derived analytically from the SG or SSG operators and the actual position of the atoms in the unit cell. These constraints are automatically kept by default during the refinements. Examples of symmetry-forced constraints generated for modulated and non-modulated magnetic structures are given in Fig. 1 ▸.

The magnetic space and superspace groups provide a concise and robust description of the magnetic ordering in periodic and aperiodic crystals and have significant practical consequences. The symmetry operations of the magnetic (super)space group define uniquely the spin of all atoms of the magnetic orbit from one representative atom of the same orbit. This simplifies equations (3) and (5) to summations only over the symmetrically independent magnetic atoms. Further, using symmetry in reciprocal space to merge symmetrically equivalent reflections improves the stability of refinements. Another important point is that macroscopic physical properties of a magnetic phase can be inferred from the symmetry operations that compose its magnetic point group, for which the respective tensors are to be kept invariant by virtue of the von Neuman principle. That is, magnetic point groups play a key role when investigating physical phenomena related to magnetic properties of matter, such as ferroelectricity. Last, but not least, the MSG and MSSG can be used to derive systematic absence conditions, which sometimes greatly help in deciding the correct magnetic model.

## 4. Symmetry of magnetic diffraction: magnetic systematic absences

We have seen that identifying the MSG or MSSG of some magnetically ordered phase in a crystal is essential for its characterization. The assignment of an MSG reduces the calculation of the magnetic moments of all symmetry-related atoms in the same orbit to only one selected representative position following the expression (9). The invariance of the density distribution function under the symmetry operation (8) can be written as:
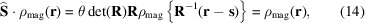
which yields
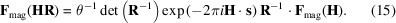


Further, the intensity of the symmetry-related point, according to (8), is
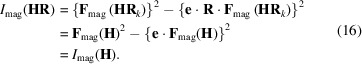


This result expresses that each symmetry operation of the point group induces rotation symmetry (proper or improper) in the diffraction pattern. An inversion center is always present in magnetic (and nuclear) diffraction, even for non-centrosymmetric groups. That is, a magnetic diffraction pattern follows classical Laue symmetry and there is no direct influence of the time inversion operation on the diffraction pattern. Consequently, the observed diffraction symmetry cannot be used directly to select the actual magnetic symmetry of the structure from the list of possible MSG.

For the case of an invariant subspace of the reciprocal space containing all vectors **H** fixed by **HR** = **H**, equation (15) leads to
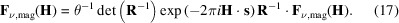


A similar expression for the scalar nuclear density directly yields systematic absence conditions linked to the factor exp(−2*πi***H** · **s**). Considering that the magnetic moment in the magnetic structure factor is a vector quantity, three equations are derived and must be fulfilled simultaneously. This fact substantially reduces the possibility of magnetic systematic absences. Yet, for translations (cell centerings), equation (17) splits into three different conditions for each component, with such systematic absences directly associated with the factor exp(−2*πi***H** · **s**).

Analogous expressions to diffraction symmetry (16) and systematic absence conditions (17) derived for **k** = 0 can be deduced for modulated structures. The differences are that a generalized magnetization density is used, and the diffraction vector is **Q** = **H** ± *m***k** (*m* > 0). All other symmetry considerations made above are valid also for the MSSGs.

Magnetic systematic absence conditions can help decide between the possible magnetic models. In *JANA2020*, the magnetic absences are derived analytically, together with all symmetry restrictions, as mentioned before. The tool MAGNEXT in the Bilbao Crystallographic Server provides the systematic absence rules for non-polarized neutron magnetic diffraction corresponding to any Shubnikov MSG in standard and non-standard settings (Gallego *et al.*, 2012 ▸; Perez-Mato *et al.*, 2015 ▸).

## 5. Representation analysis in *JANA2020*

The two approaches that have been historically most used to describe and analyze magnetic structures were briefly presented in the *Introduction*[Sec sec1]. Group-representational theory (Bertaut, 1968 ▸) and magnetic symmetric descriptions of a magnetically ordered phase are not generally equivalent, as there is only a one-to-one correspondence between a one-dimensional real irrep of the parent space group and a magnetic space group of the same geometry class (Niggli, 1959 ▸; Indenbom, 1959 ▸).

A combined approach was developed by Stokes & Hatch (1988 ▸) and consists in calculating the possible MSGs (or MSSGs) for the set of active irreps from the paramagnetic phase, the so-called isotropy subgroups of an irrep. These subgroups necessarily contain the symmetry operators that verify the invariance equation for the irrep. For any single-irrep mT of a non-modulated magnetic structure, this can be written as:
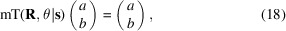
where mT (**R**, θ | **s**) is the matrix of a physically irreducible magnetic representation, and *a*,*b* arbitrary real numbers. The symmetry operations of the gray space group that keep the vector (*a*,*b*) invariant and transform under the irrep as unit matrices form the so-called kernel of the irrep. That is, the kernel symmetry of an irrep is the isotropic subgroups corresponding to most general directions (*a*,*b*) in the irrep space (or order parameter direction, OPD). Isotropy subgroups corresponding to symmetry operations that fulfill equation (18) for specific order parameter directions are called epikernels and correspond to allowed symmetries higher than the kernel.

Representation analysis of incommensurate (single-*k*) modulated structures departing from the representations of the paramagnetic space group follows the principles explained above with a few details. In this case, the paramagnetic phases are given by irreps containing the pure time inversion operation **Ŝ**_s_ = (**E**, −1 | 0, 0, 0, 0) defined in the respective four-dimensional space. Any irrep corresponding to a magnetic order parameter associates the unit matrix combined with time inversion and necessarily includes the operation **Ŝ**_s_ = (**E**, −1 | 0, 0, 0, ½). This is a general property of any single-*k* incommensurate magnetic modulation, which stems from the harmonic character of any fundamental modulation and satisfies the invariance equation for the symmetry operations of the magnetic superspace group [analogous to equation (18), see *e.g*. Perez-Mato *et al.*, 2012 ▸). It is evident that a π phase shift combined with the action of the pure time inversion operator keeps the system invariant. One of the consequences of this operation is that the Fourier terms in (4) are constrained to odd-order harmonics, whereas even-terms are allowed for any structural modulations. Additionally, the presence of this operation means that the phase has a gray point group. That is, linear magneto-structural couplings are not possible, and ferromagnetism (or ferrotoroidicity) is forbidden for single-*k* incommensurate phases. This simple property emphasizes the importance and robustness of magnetic symmetry considerations in predicting and understanding symmetry-related properties of phases.

It has been shown that the direct use of MSGs or MSSGs simplifies the algorithms of programs refining experimental data, whereas the selection of an irrep might provide extra relationships which improve the efficiency and stability of the programs dealing with magnetic diffraction data (Petříček *et al.*, 2010 ▸).

**Figure 2:**
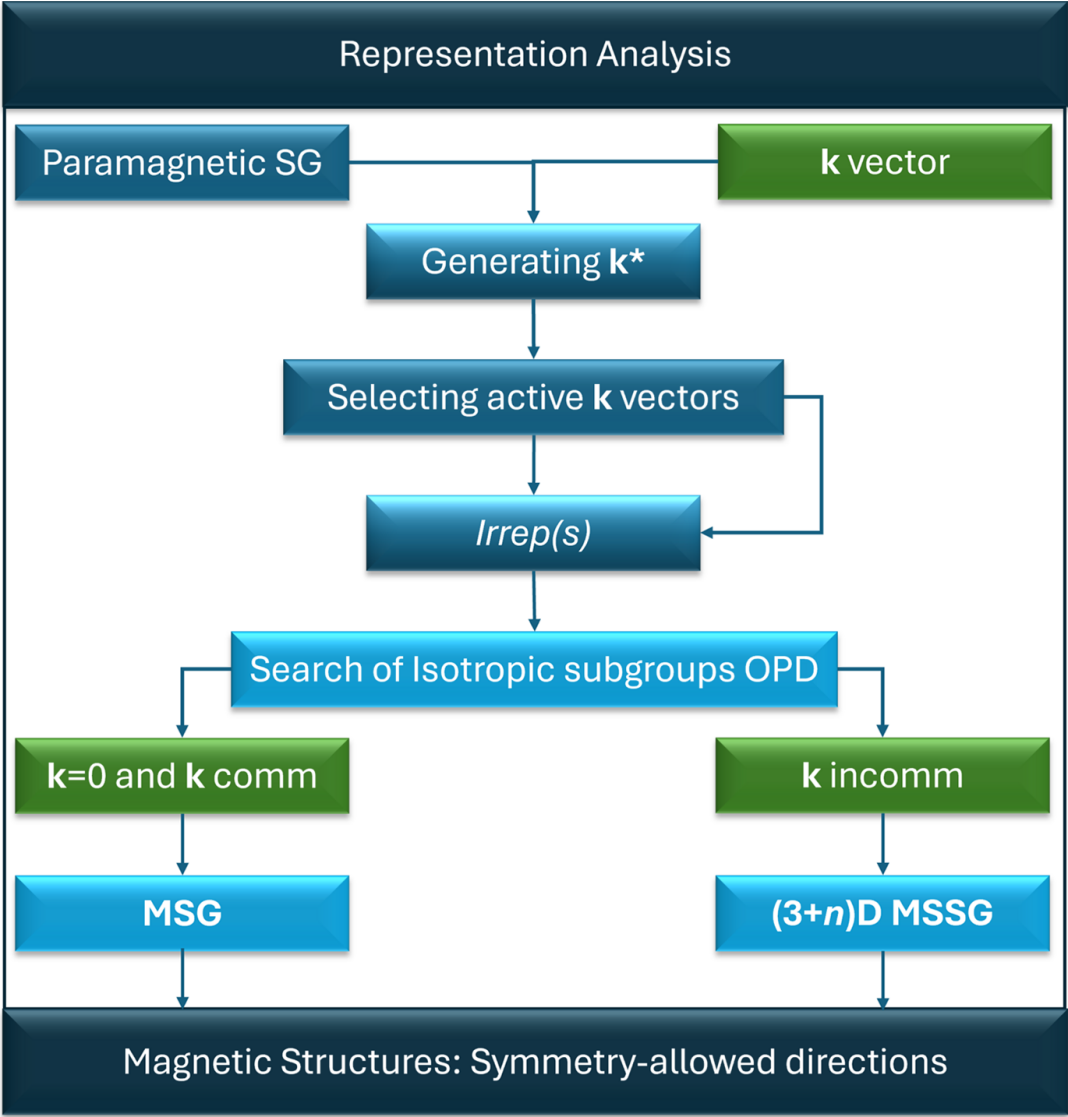
Scheme of the combined approach used in *JANA2020* for representation analysis. This method allows the straightforward description of all types of magnetic structures (modulated or not) using magnetic crystallography and departing from the possible active irreps of the paramagnetic space group and the respective propagation vector. The k* refers to the set of distinct **k** vectors related by the symmetry of the (parent) space group. The terms ‘**k** comm’ and ‘**k** incomm’ signify the propagation vectors of commensurate and incommensurate structures, respectively, as defined in the *Introduction*[Sec sec1].

The combined approach described here is implemented in the magnetic option of *JANA2020*. For a single irrep, the current version of the program allows full representation analysis except for (3+*n*)-dimensional MSSGs for *n* > 1. Moreover, the possibility of combining irreps is also under development. The embedded tool of *JANA2020* first produces listings of irreps with the respective basis vectors. Then, it calculates the set of MS(S)Gs that are the isotropy subgroups for the kernel (general OPD) and epikernel (specific OPD) symmetries of the active irreps for the space group of the paramagnetic phase. The magnetic structures are then the result of the configuration of spins in directions admissible by the MSG or MSSG. This methodology is presented in Fig. 2 ▸ for all types of magnetic propagation vectors.

For the case of multi-*k* modulated structures, magCIF files are prepared in *JANA2020* and then imported into *ISODISTORT* (Campbell *et al.*, 2006 ▸; Stokes *et al.*, 2023 ▸) for representation analysis. This program calculates the kernel and epikernels of any possible irrep (one or more) of the parent space group symmetry independently of the dimension of the modulation. The resulting models can be saved as magCIF files and then imported and tested in *JANA2020* against diffraction data.

## 6. The magnetic option in *JANA2020*: features and workflow

**Figure 3:**
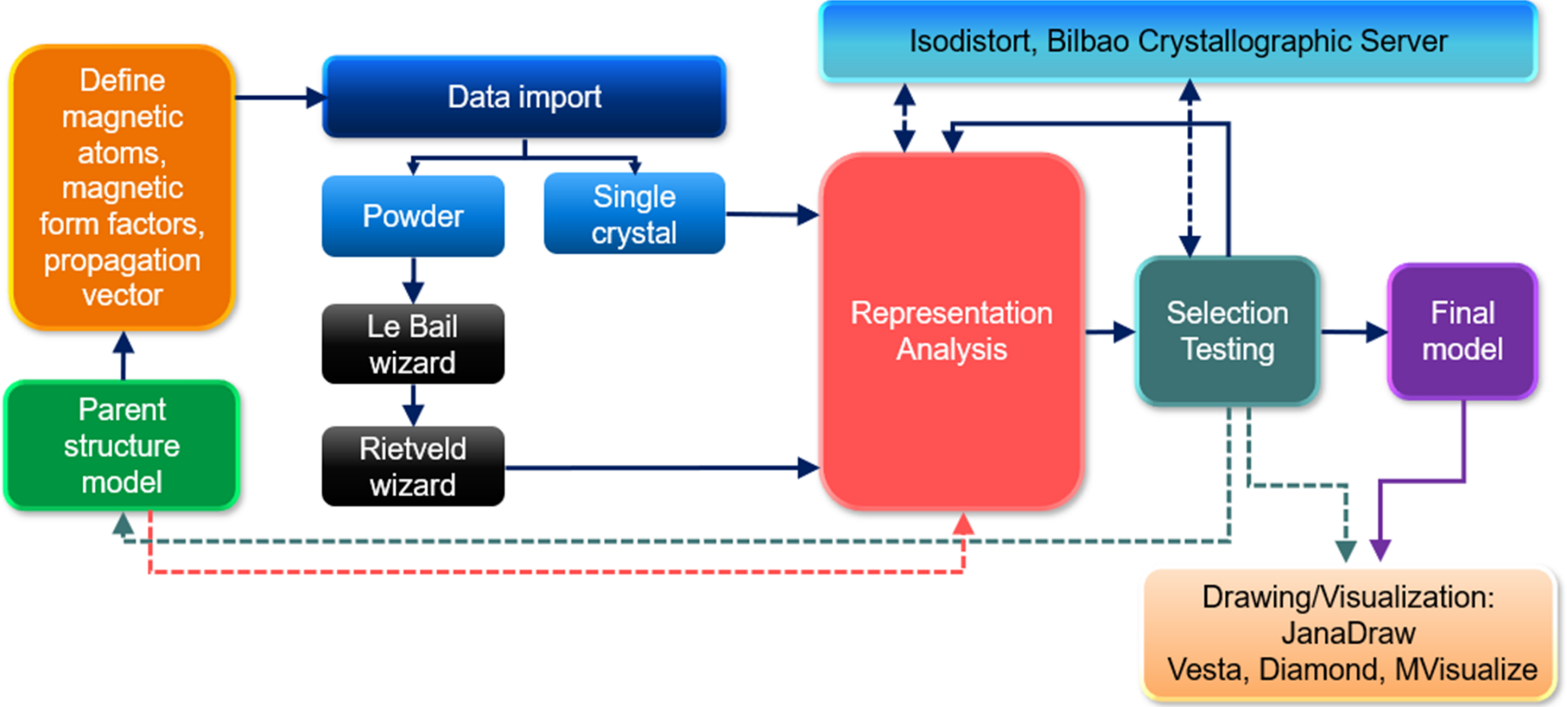
Workflow scheme of the magnetic wizard in *JANA2020*, showing the main procedure with dark-blue continuous arrows and other parallel path options linked by colored discontinuous arrows.

**Figure 4:**
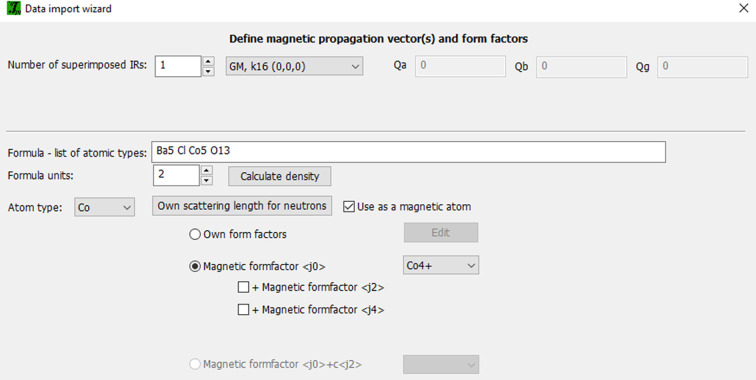
Interface for defining the basic magnetic parameters of the parent structure model. The number and type of irrep for a structure can be conveniently selected from a drop-down menu listing all possible representations in CDML (Cracknell *et al.*, 1979 ▸) and Kovalev (1993 ▸) notations.

The solution and refinement of magnetic structures in *JANA2020* follows the basic scheme presented in Fig. 3 ▸. The first step is defining the parent structure. It can be imported (from Crystallographic Information Files (CIF), *JANA* files or structural files of other programs) into the wizard and completed with specific magnetic details: type and number of irreps or form of the magnetic propagation vector(s), magnetic atoms present in the structure, and their respective magnetic form factors. All this information is entered into a single window shown in Fig. 4 ▸ and saved. Then, the neutron diffraction data files are imported into *JANA* by using one of the pre-defined formats covering most neutron instruments (constant wavelength or time-of-flight data) or by reading a general format reflection file. For powder samples, the program guides the user through refinements of the profile parameters (le Bail) and the scale factor (Rietveld). The parent structure created in the first stage is fixed and used to perform representation analysis, which is the core of magnetic structure determination (see Fig. 3 ▸).

**Figure 5:**
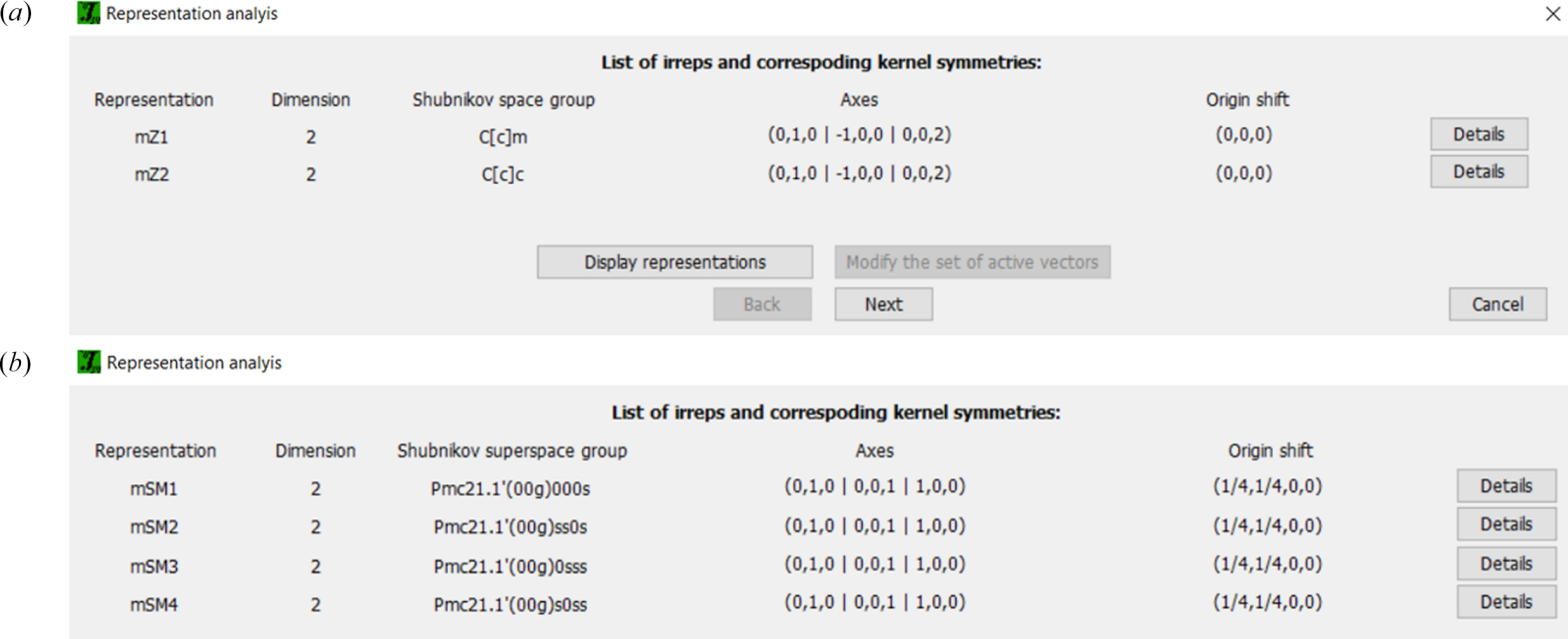
First informative window after representation analysis for a (*a*) parent space group *Cmcm* and **k** = (00½) and (*b*) parent space group *Pnma* and **k** = (α00). It lists the minimal symmetry groups (kernels) for the correspondent irreps. The lateral button ‘Details’ displays the symmetry operators for the kernel space group (*a*) or superspace group (*b*) of the irrep and the respective transformation to the standard setting. The irrep matrices can be consulted in ‘Display representations’ at the bottom.

**Figure 6:**
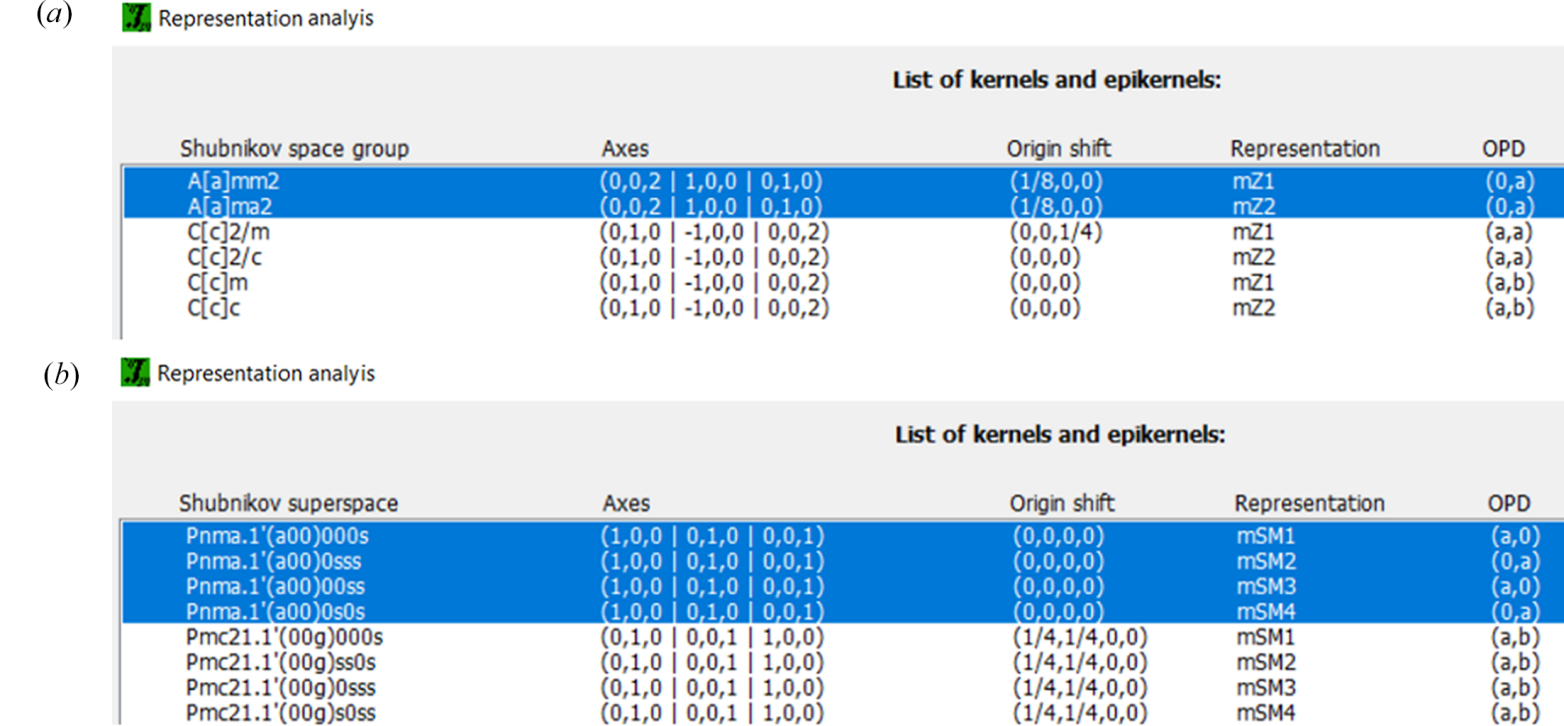
Second representation analysis window listing the isotropy subgroups associated with the irreps and respective order parameter directions (OPD) for structures (*a*) commensurate and (*b*) incommensurate from Fig. 5 ▸.

*JANA* starts the representation analysis using irrep matrices from Stokes *et al.* (2013 ▸). It first displays an informative window listing the irreps of the structure and their corresponding kernel subgroups, as presented in Fig. 5 ▸. Next, the program offers a list of all kernels and epikernels grouped in blocks ordered from higher to lower symmetry (Fig. 6 ▸). Each block corresponds to the possible combinations of the (nuclear) space group operators with time inversion for MSGs and with the internal translational components for MSSGs.

**Figure 7:**
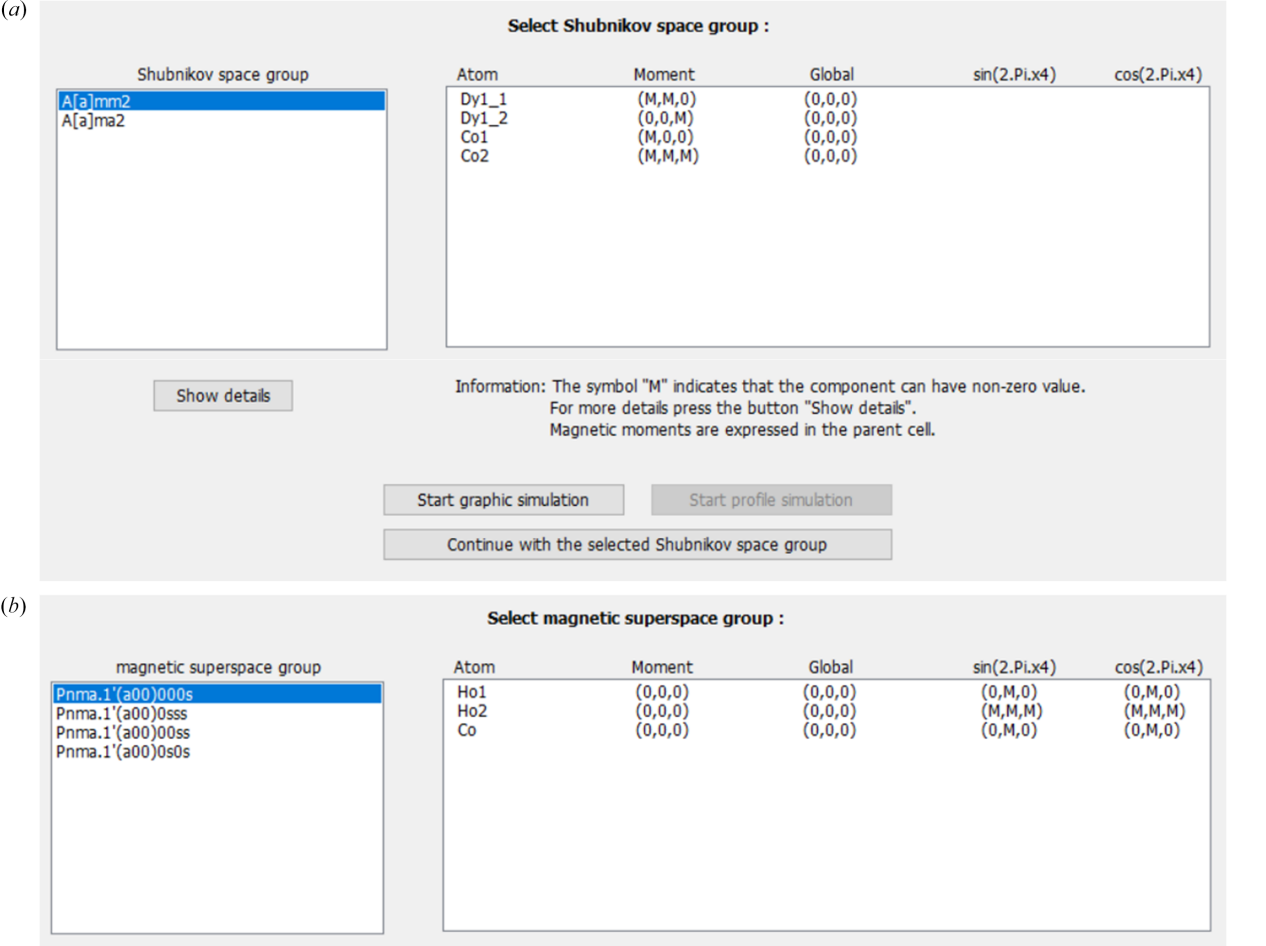
Third step of representation analysis: testing and selecting promising magnetic models for refinement. Detailed information about each model is given in this window, such as the symmetry restrictions over the moment components (*M*) for (*a*) commensurate and (*b*) incommensurate structures from Figs. 5 ▸ and 6 ▸. Simulation of magnetic arrangements and powder profiles for the offered space groups is also possible here for single crystals and powders, respectively.

Detailed information about the magnetic model for each specific space (or superspace) group in the selected set of subgroups is subsequently given (Fig. 7 ▸). Here, the user can select and inspect the models by checking the symmetry-allowed components for the moments in the structure, global moment, or induced orbit splitting. Complete information about the symmetry restrictions can be found in ‘Show details’.

For powders, testing can be done by comparing the experimental powder pattern against a profile simulation for random magnetic moments by ‘Starting profile simulation’. For powders and single crystals, the button ‘Start graphic simulation’ produces a graphical preview of randomly generated magnetic moment vectors in a unit cell. These are both very useful tools to conveniently reduce the number of models to be refined against data: the first by making direct use of magnetic systematic absences; the second by checking spin configurations that, for example, are not compatible with macroscopic properties of the crystal.

Finally (or not!), the promising symmetry(ies) can be selected for refinement. To this, *JANA2020* launches the refinement separately for each model with a different job name, keeping the window with the parent structure always available for further model trials or other tasks.

When there is a need to perform representation analysis in *ISODISTORT*, *JANA2020* connects it directly by ‘Run *ISODISTORT* analysis’ accessible from the parent structure. At this point, JANA automatically prepares the CIF of the parent structure and instructs the user on how to proceed. The output of *ISODISTORT* can be saved as an individual magCIF. These files are then imported into *JANA*, and the testing/selection steps proceed as mentioned above.

For visualization of the magnetic models, the new intuitive plotting tool *JANADraw* inspects in real-time the changes in the magnetic structure throughout refinements. *JANA* can also directly call the program *VESTA* (Momma & Izumi, 2011 ▸). Nevertheless, this program plots only approximants for modulated structures, whereas the plots of *JANADraw* can precisely reproduce the complete modulation by changing the internal space coordinate (see below).

The internal coordinate *x*_4_ introduced in (13) is conveniently changed to *t* which characterizes the real three-dimensional structure,
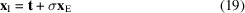
where **x**_I_ and **x**_E_ are the internal and external coordinates, respectively, **t** is the initial phase of the modulation, and σ is the matrix containing the components of the modulation vector(s). In fact, the *t*-sections are perpendicular to the internal subspace of the superspace.

For a general structural modulation, the user of *JANA2020* can choose to plot the modulated parameter as function of either *x*_4_ or *t*. In the case of modulated magnetic structures, the amplitude of the magnetic moment(s) varies as a function of the internal space coordinate **t** or **x**_I_. However, such graphs will be shifted by the phase factor σ**x**_E_. To compare more modulation functions by drawing them into one figure only the *t*-plot can be used as it is related to the atomic parameters as they really exist in physical space. This is also true for any characteristic in which two or more atomic parameters are combined (distances, angles, *etc*.).

**Figure 8:**
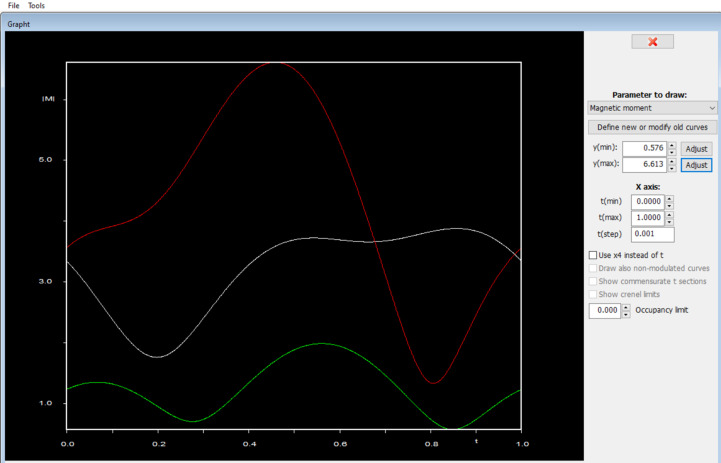
*Grapht* plot of the magnetic moment amplitude (in μ_B_) variation as a function of the internal coordinate *t* (horizontal axis) for three different magnetic atoms in the incommensurate structure from Figs. 5 ▸–7 ▸ ▸.

In *JANA*, the amplitude change of the magnetic moment can be plotted in a tool called *Grapht* (see Fig. 8 ▸). *JANADraw* can also produce an ‘animated’ model of the modulated structure as a function of **t**. This movie helps to visualize and describe details of the spin modulation, such as type of arrangement, spin directions and angles, and propagation direction.

**Figure 9:**
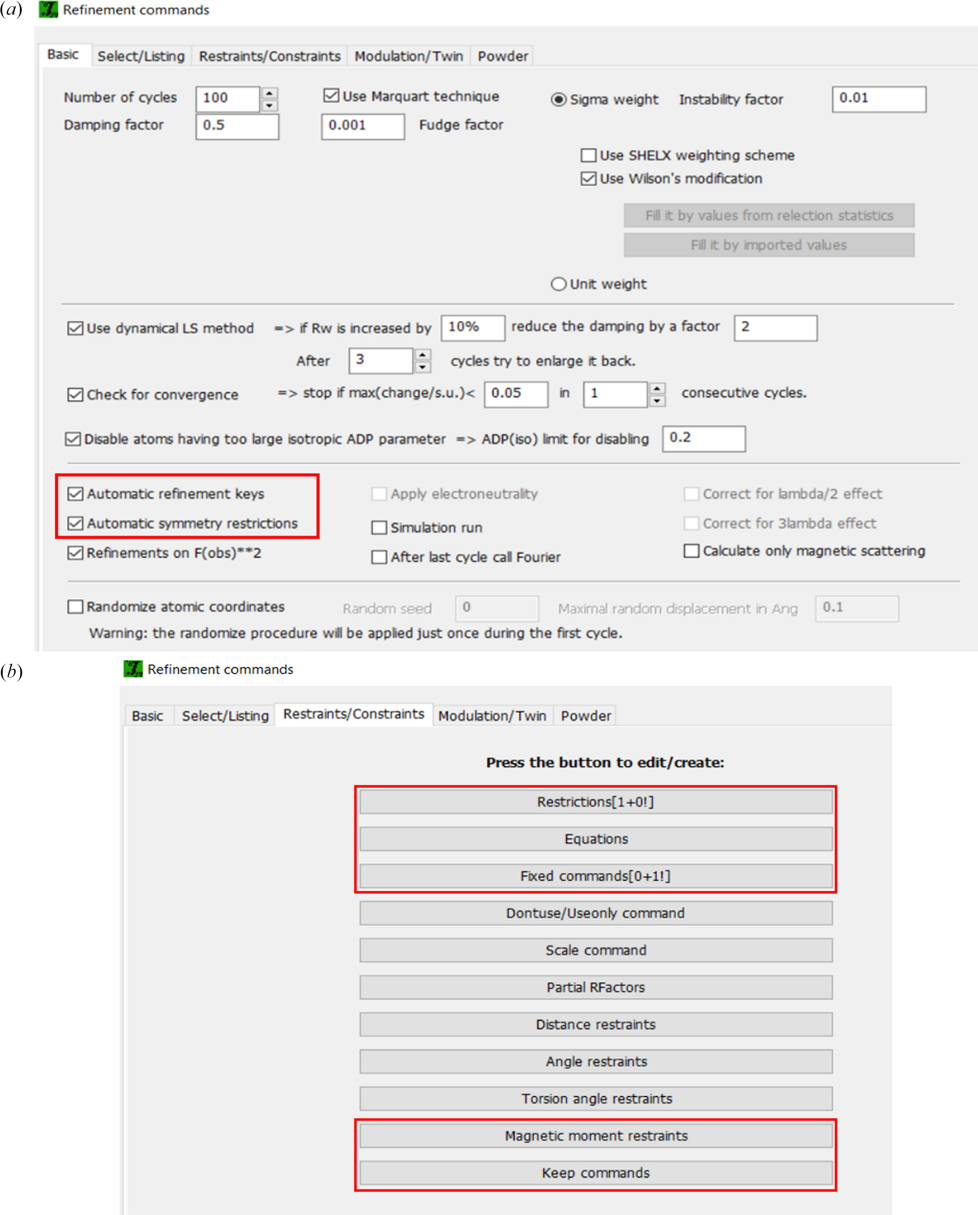
Different options under ‘Refinement commands’: (*a*) automatically generated symmetry restrictions are used by *JANA* throughout the refinements; (*b*) individual parameters can be edited or user-defined in several forms. The buttons highlighted in red can control magnetic-moment-related parameters.

As already mentioned, *JANA2020* derives all symmetry constraints analytically and keeps them active during the refinements as automatic keys [see Fig. 9 ▸(*a*)]. When refining magnetic models, *JANA* also automatically fixes the structural parameters (atomic positions and thermal parameters). This constraint and any other restrictions over refinement parameters are available for editing on the page ‘Restraints/Constraints’ [Fig. 9 ▸(*b*)].

The ‘Restrictions’ commands refer to selected parameters of atoms that are made identical or related by a symmetry operation. Here, it is possible, *e.g.* to make identical magnetic parameters for the specified atoms. In ‘Equations’, user-defined linear equations containing combinations of any parameters can be set. In *JANA*, each variable has an identifying name that can be used to write the equations for the model. The option ‘Magnetic moment restrains’ allows us to fix the magnitude of the spin of an atom to a specific value or to keep the magnitude equal for a certain group of atoms. For modulated magnetic structures, the magnetic moment amplitude can be fixed to an optimal value, allowing only the orientation to be affected by the modulation. In ‘Keep commands’, the geometry of a cyclo­idal modulation can be kept for selected atoms.

*JANA2020* magnetic option also offers the possibility of transforming the magnetic structure model. The most useful pre-defined transformations are group-subgroup transformation, origin shift, and transformation to standard-setting. The ‘Go to Subgroup’ tool is used to reach subgroups of any selected space group, for example, when there are zero and non-zero **k** vectors associated, or specific magnetic reflections not explained by the groups of higher (maximal) symmetry. The interactive wizard first lists all the symmetry operations in the group, from which the user can select the one(s) to be present in the subgroup. Once the representatives are selected, the program completes the subgroup. The original MSG or MSSG breaks up into individual cosets, from which the program automatically chooses one symmetry element as ‘coset representative’ for each coset. These representatives generate the new atomic positions and twin matrices when applicable. It can be defined manually or automatically. The new output structure is saved separately and is ready for testing.

**Figure 10:**
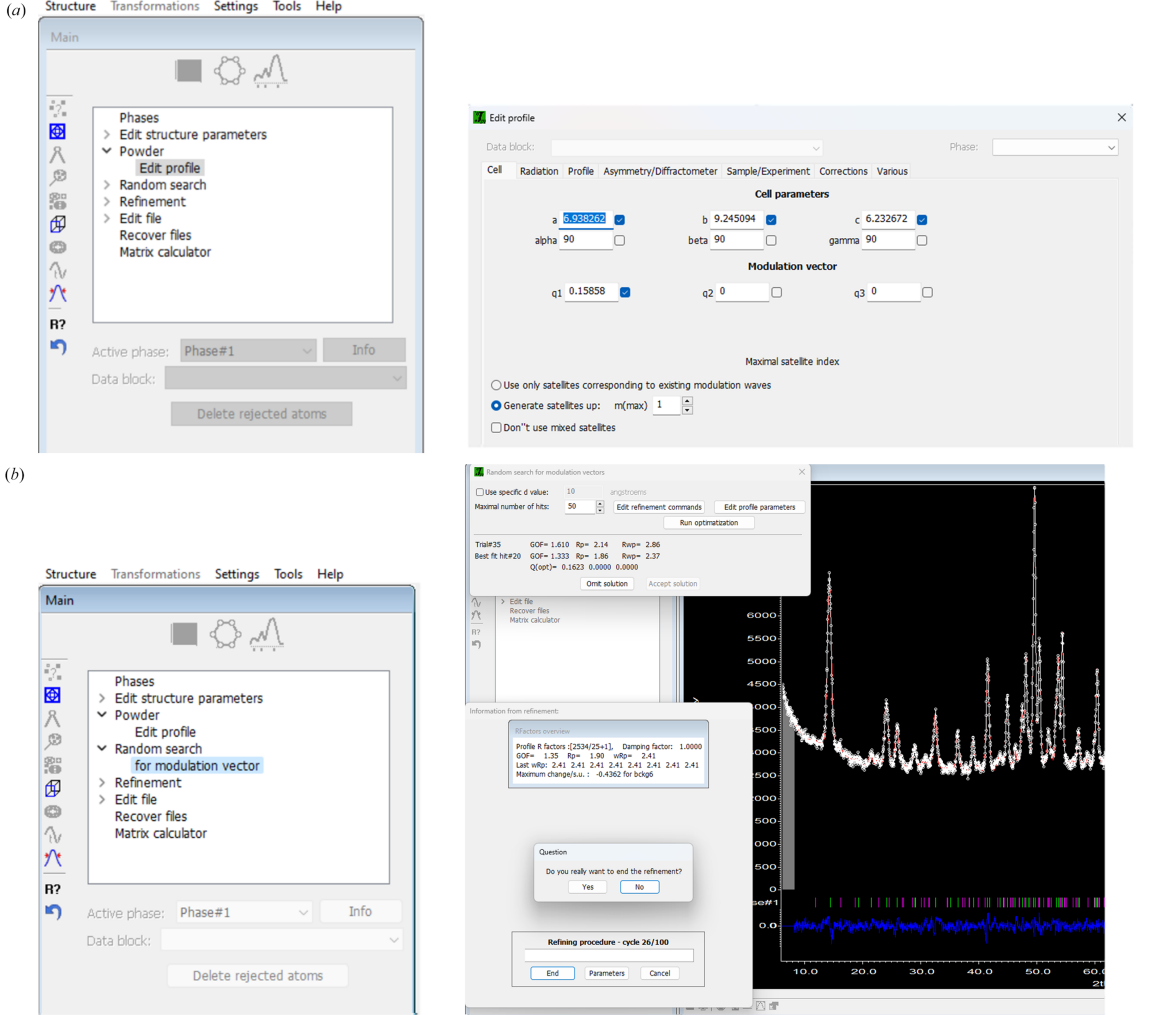
Sequence to make a random search for the **k** vector for powder data.

**Figure 11:**
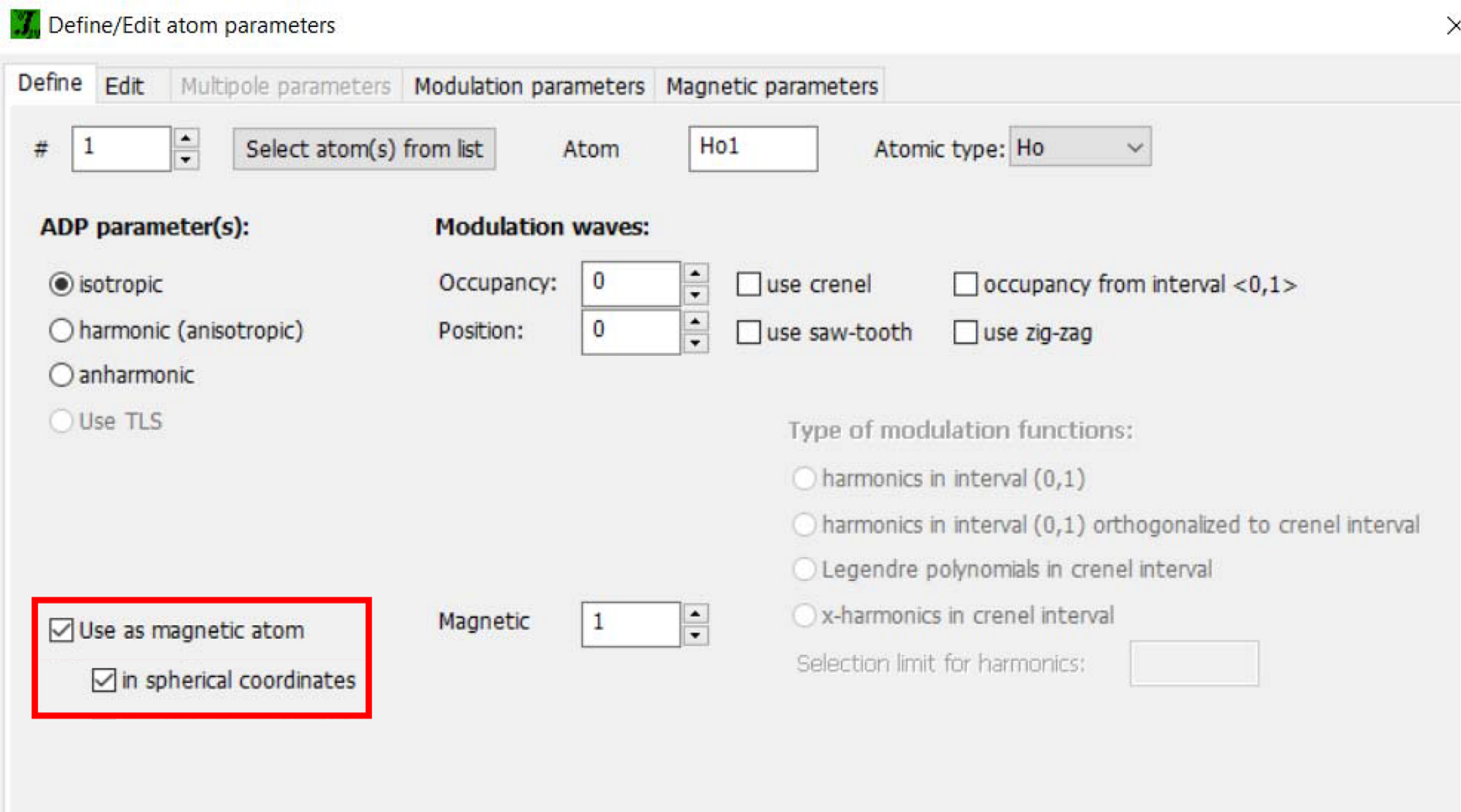
In the magnetic wizard of *JANA2020*, the magnetic moments can be refined in spherical coordinates as an alternative to the Cartesian coordinate system.

Other tools specific to the magnetic option in *JANA2020* include the possibility of searching for the **k** vector for powder data and refining magnetic moments in spherical coordinates. The random search for the **k** vector is accessible from the parent phase. The user must change the type of propagation vector in ‘Edit profile parameters’, which activates the option ‘Random search for modulation vector’ as shown in Fig. 10 ▸. The option to have the magnetic moments in spherical coordinates is available when editing the magnetic parameters of the atoms (Fig. 11 ▸). For the two angles and the amplitude of the moment no symmetry restrictions are derived automatically and must be manually added in ‘Equations’.

## 7. Examples

Two examples of magnetic structures solved in the magnetic option of *JANA2020* are presented and discussed here to illustrate the operation and capabilities of the program.

### 7.1. The commensurate magnetic structure of Dy_2_Co_3_Al_9_ at 2 K

The compound Dy_2_Co_3_Al_9_ has interesting electronic properties linked to exchange frustration. It undergoes three consecutive antiferromagnetic transitions, including one of first order at *T*_N_ = 3.7 K (Gorbunov *et al.*, 2018 ▸). It crystallizes in a layered orthorhombic structure (space group *Cmcm*), in which the Dy atoms occupy the 8*g* site (*x*, *y*, ¼) and are arranged in a hexagonal network. The Co atoms are in two different positions (8*e*, 4*a*) and Al occupies four independent positions (4*c*, 8*f*, 8*g* and 16*h*). Refined coordinates can be found in Gorbunov *et al.*, 2018 ▸.

The magnetic phase for *T* < 3.7 K was probed by single-crystal neutron diffraction at 2 K. The magnetic propagation vector for this phase was found to be **k** = (0 0 ½), that is the magnetic cell is doubled along the **c** direction compared to the crystallographic cell.

Representation analysis performed in *JANA2020* indicates two two-dimensional irreps for this **k** vector (point *Z* in the Brillouin zone): mZ1 and mZ2. Their respective matrices are given in Table 1 ▸. The corresponding irrep kernels and epikernels returned in the analysis [see Figs. 5 ▸(*a*) and 6 ▸(*a*)] are given in Table 2 ▸.

There are four epikernels corresponding to the four maximal space groups of the gray group *Cmcm*1′. From the refinement of the experimental data, it follows that the magnetic structure of Dy_2_Co_3_Al_9_ is described by the MSG *A_a_mm*2 corresponding to the irrep mZ1 but restricted to a special direction in the irrep space. The symmetry operations of this MSG are listed in Table 3 ▸.

**Figure 12:**
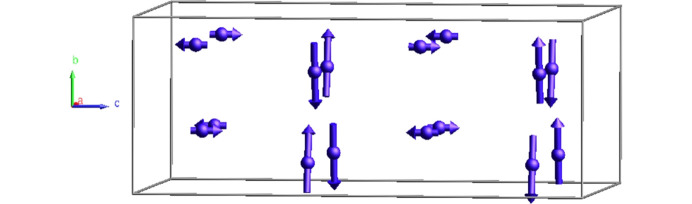
The magnetic structure of Dy_2_Co_3_Al_9_ at 2 K is described in the non-centrosymmetric MSG *A_a_mm*2. The structure consists of alternating layers of moments oriented parallel to (110) and moments along [001]. Only the Dy atoms are represented in the unit cell.

The point group symmetry of the magnetic arrangement of Dy_2_Co_3_Al_9_ is *mm*21′. Note that due to the lattice translations, the symmetry operator {1′ | 0 0 ½} belongs to the point group of the magnetic phase (see Table 3 ▸). This is a polar point group, which means that macroscopic polarization is allowed for this phase by the MSG. The decrease of point group symmetry splits the Dy 8*g* position into two independent magnetic orbits with different degrees of freedom. One of the positions allows noncollinear magnetic moments parallel to the *ab* plane, whereas the other restricts the magnetic moments to be collinear and oriented along the *c* axis (see Table 4 ▸). The magnetic unit cell of Dy_2_Co_3_Al_9_ is obtained by alternating these two configurations along the *c* axis, as shown in Fig. 12 ▸. The larger Dy moment is found along the *b* axis. It amounts to 8.4 (1) μ_B_/Dy atom and lies at angles of ±81 (1)° away from the *a* axis. All the free parameters and refined values are specified in Table 4 ▸. With the symmetry reduction, two equally populated twin domains are automatically created.

As the MSG is a *k*-maximal subgroup, the spin ordering is only allowed according to a single irrep, further restricted to fulfill the MSG constraints. So, there is no advantage in using irrep analysis alone. This structure is entry No. 1.267 in MAGNDATA (Gallego *et al.*, 2016*a* ▸,*b* ▸).

### 7.2. The incommensurate magnetic structure of Ho_3_Co at 18 K

The rare earth-rich intermetallic compound Ho_3_Co crystallizes in Fe_3_C-type orthorhombic structure with *Pnma* space group (Baranov *et al.*, 2005 ▸; Podlesnyak *et al.*, 2004 ▸). In this crystal structure, Ho has two crystallographic positions [4*c* (*x*, ¼, *z*) for Ho1 and 8*d* (*x*, *y*, *z*) for Ho2] that form trigonal prisms within which the transition metal Co shares the same Wyckoff position as Ho1.

Ho_3_Co is an interesting case, as it exhibits two different antiferromagnetic transitions at different temperatures: one below *T*_N_ (∼21–22 K) and the other below *T*_T_ (∼8–9 K) (Baranov *et al.*, 2005 ▸; Podlesnyak *et al.*, 2004 ▸; Goswami *et al.*, 2020 ▸). The magnetic structures of the phases are unknown.

To solve the magnetic structure of Ho_3_Co, neutron powder diffraction was performed at 18 K. The powder pattern was refined in *JANA2020* (Petříček *et al.*, 2014 ▸, 2023 ▸). After refining the profile parameters (LeBail), a search for a suitable modulation vector was performed through the random search procedure in *JANA2020* (see Fig. 10 ▸). The magnetic propagation vector was found to be **k** = [0.1585 (19), 0, 0].

Representation analysis was executed using the magnetic option of *JANA2020*. This step provided a list of MSSGs consistent with the possible irreps for the parent structure (gray magnetic group *Pnma*1′) for the propagation vector **k**. This propagation vector lies in the Σ-line of the Brillouin zone and the magnetic atoms in this compound reside at the same positions as the atoms of the paramagnetic crystal structure. Thus, there are four possible two-dimensional (small) irreps: mSM1, mSM2, mSM3 and mSM4. The corresponding ortho­rhombic MSSGs associated with the kernels and epikernels of these irreps, the respective order parameter direction (OPD), and the number of free parameters imposed by symmetry are listed in Table 5 ▸.

The MSSG *Pnma*.1′(α00)0*s*0*s* (mSM4) provided the best fit among these possibilities. Yet, the calculated intensities for the nuclear reflections did not match the experimental ones without adding a magnetic contribution from a **k**_0_ component. Thus, the time-reversal operation must be removed from the set of operations describing the structure. As a result, the magnetic symmetry is transformed to a subgroup of the MSSG *Pnma*.1′(α00)0*s*0*s* with non-gray point-group symmetry.

The four MSSGs fulfilling this criterion are *Pn*′*m*′*a*(α00)000, *Pnm*′*a*′(α00)000, *Pn*′*m*′*a*′(α00)000, and *Pnm*′*a*(α00)000. Each transformed MSSG corresponds to a set of active irreps that includes the contribution coming from the Γ-point (GM) and from the Σ-line (SM) modes. The possible active irreps are now mGM2+ | mSM4, mGM3+ | mSM4, mGM1− | mSM4, and mGM4− | mSM4. The experimental data are best fit by the MSSG *Pnm*′*a*(α00)000 (No. 62.444) and the corresponding irreps mGM4− | mSM4 with OPD (*a* | *b*, 0). Both mGM4− and mSM4 act as primary irreps in this magnetic structure. The small irrep mGM4− is one-dimensional, and thus, the matrix representations of the combined irreps that provide the final solution for the magnetic structure of Ho_3_Co are shown in Table 6 ▸. The symmetry associated with this MSSG is given in Table 7 ▸.

Based on the symmetry as shown in Table 7 ▸, any operation such as 2_1_′ [0, 0, 1] provides the relation between the modulation functions of the magnetic moments as *M* (*x*_4_ + ½) = 2_1_′.*M*(*x*_4_). Thus, by considering the expression (13), we have *M_i_*(*x*_4_ + ½) = −*M*_i, sin_sin(2π*nx*_4_) −*M*_i, cos_cos(2π*nx*_4_), where *i* = *x*, *y*, *z* and *M*_*i*,sin1_ = −*M*_*i*,sin1_ and *M*_i,cos1_ = −*M*_i,cos1_. Note that because this structure is described by a single **k** incommensurate vector (without higher order harmonics), the summation (14) runs only for *n* = 1, yielding ‘sin1’ and ‘cos1’ for the respective terms of the modulated magnetization (first, main) wave of each magnetic atom type present. Combining these results with the symmetry constraints, the possible components for each magnetic atom are obtained (Table 8 ▸).

**Figure 13:**
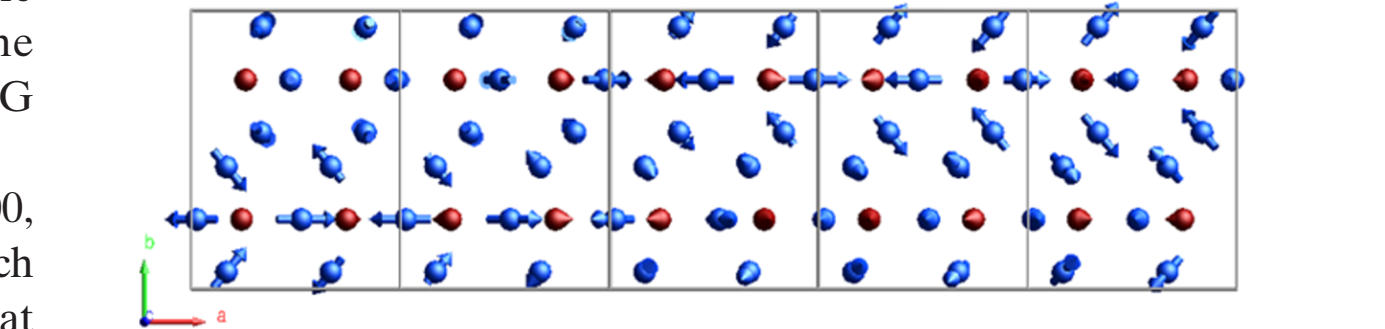
Spin modulation of Ho_3_Co at 18 K for Ho (blue) and Co (red) represented in a 5 × 1 × 1 supercell of the parent orthorhombic unit cell. The modulation of Ho1 and Ho2 is described as fanlike, with a fan spanning of 100°. The fan plane of the Ho1 is parallel to (101), whereas Ho2 spins are at an angle of 57° away from the *a* axis. The modulation of the Co spins is a circular cycloidal restricted to be parallel to (101).

The refined magnetic model is visualized in *JANADraw* and presented in Fig. 13 ▸. The magnetic point group is *m′mm*, with the Ho1 and Co spins moving only parallel to (101). The modulation of the Ho1 spins can be described as fanlike (Buschow, 1977 ▸), where the fan has a span of ∼100° with a moment amplitude of 4.1 (3) μ_B_. The neighboring spin along the modulation direction has a reverse sense. Ho2, on the other hand, is at the general 8*d* position, with free spin components in all space directions. Its modulation is also fanlike, but in a plane of about 57° away from [100]. The fan span for Ho2 is approximately 100°, but because of the twofold improper rotation along the modulation direction, there is a shift in the plane and sense of the spin. The maximal moment amplitude of Ho2 is 2.72 (10) μ_B_. The transition metal Co also carries a magnetic moment with a maximal value of 1.4 (3) μ_B_ and describes a circular cycloid. The individual modulations of Ho1, Ho2, and Co are shown in Fig. 8 ▸ as plotted by *Grapht* as red, white, and green waves, respectively.

## Figures and Tables

**Table 1:** Irrep matrices defining the magnetic space group *Cmcm*1′ with wavevector **k** = (0 0 ½), point *Z* in the Brillouin zone

	1 [0, 0, 0]	2_1_ [0, 0, 1]	2 [0, 1, 0]	2 [1, 0, 0]	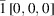	m [0, 0, 1]	m [1, 0, 0]	*c* [0, 1, 0]	1′
mZ1	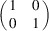	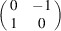	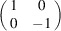	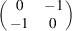	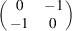	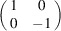	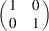	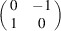	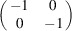
mZ2	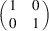	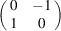	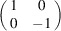	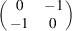	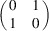	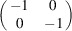	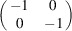	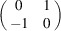	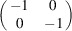

**Table 2:** Epikernels and kernels of the magnetic irreps of *Cmcm*1′ at the point *Z*

Irrep	OPD	MSG	Free parameters
mZ1	(0, *a*)	*A_a_mm*2	3
	(*a*, *a*)	*C_c_*2/*m*	3
	(*a*, *b*)	*C_c_m*	6
mZ2	(0, *a*)	*A_a_ma*2	3
	(*a*, *a*)	*C_c_*2/*c*	3
	(*a*, *b*)	*C_c_c*	6

**Table 3:** Symmetry operations defining the magnetic structure of Dy_2_Co_3_Al_9_ at 1.5 K in the MSG *A_a_mm*2

{1 | 0 0 0}	*x*	*y*	*z*	*m*
{2_010_ | 0 0 ¾}	−*x*	*y*	−*z* + ¾	*m*
{*m*_100_ | 0 0 0}	−*x*	*y*	*z*	*m*
{*m*_001_ | 0 0 ¾}	*x*	*y*	−*z* + ¾	*m*
{1′ | 0 0 ½}	*x*	*y*	*z* + ½	−*m*
{2′_010_ | 0 0 ¾}	−*x*	*y*	−*z* + ¼	−*m*
{*m*′_100_ | 0 0 ½}	−*x*	*y*	*z* + ½	−*m*
{*m*′_001_ | 0 0 ¾}	*x*	*y*	−*z* + ¼	−*m*

**Table 4:** Refined parameters for the Dy_2_Co_3_Al_9_ magnetic phase at *T* = 2 K in the Shubnikov group indicated in BNS notation (the second space group symbol is the UNI symbol of the group)

	Transformation to the standard setting	Atom label	Symmetry constraints on *M_i_*	*m_x_* (μ_B_)	*m_y_* (μ_B_)	*m_z_* (μ_B_)
***A_a_mm*2** (No. 38.192) {*Amm*2.1′*a*[*Amm*2]}	(*c*, *a*, *b*; 0, 0, − 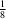 )	Dy1_1	*m_x_*, *m_y_*, 0	1.34 (2)	8.35 (2)	0
		Dy1_2	0, 0, *m_z_*	0	0	1.38 (1)

**Table 5:** MSSGs compatible with the irreps, respective OPD, and the number of free parameters to be refined in each case

Irreps	MSSG	OPD	Free parameters
mSM1	*Pnma.*1′(α00)000*s*	(*a*, 0)	2 (Ho1) + 6 (Ho2) + 2 (Co)
	*Pmc*2_1_.1′(00γ)000*s*	(*a*, *b*)	4 (Ho1) + 12 (Ho2) + 4 (Co)
mSM2	*Pnma.*1′(α00)0*sss*	(0, *a*)	4 (Ho1) + 6 (Ho2) + 4 (Co)
	*Pmc*2_1_.1′(00γ)*ss*0*s*	(*a*, *b*)	8 (Ho1) + 12 (Ho2) + 8 (Co)
mSM3	*Pnma.*1′(α00)00*ss*	(*a*, 0)	2 (Ho1) + 6 (Ho2) + 2 (Co)
	*Pmc*2_1_.1′(00γ)0*sss*	(*a*, *b*)	4 (Ho1) + 12 (Ho2) + 4 (Co)
mSM4	*Pnma.*1′(α00)0*s*0*s*	(0, *a*)	4 (Ho1) + 6 (Ho2) + 4 (Co)
	*Pmc*2_1_.1′(00γ)*s*0*ss*	(*a*, *b*)	8 (Ho1) + 12 (Ho2) + 8 (Co)

**Table 6:** Matrix representations of the irreps, along with the OPD, that provide the best solution for Ho_3_Co

	1 [0, 0, 0]	2_1_′ [0, 0, 1]	2_1_ [0, 1, 0]	2_1_′ [1, 0, 0]	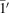 [0, 0, 0]	*a* [0, 0, 1]	*m*′ [0, 1, 0]	*n* [1, 0, 0]	
mGM4−|mSM4	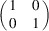	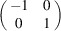	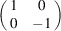	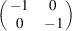	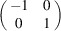	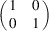	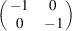	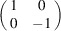	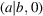

**Table 7:** Representative operations of MSSG *Pnm*′*a*(α00)000 as used in *JANA2020*

1[0,0,0]	*x*_1_	*x*_2_	*x*_3_	*x*_4_	+*m*
2_1_′[0,0,1]	−*x*_1_ + ½	−*x*_2_	*x*_3_ + ½	−*x*_4_ + ½	−*m*
2_1_[0,1,0]	−*x*_1_	*x*_2_ + ½	−*x*_3_	−*x*_4_ + ½	+*m*
2_1_′[1,0,0]	*x*_1_ + ½	−*x*_2_ + ½	−*x*_3_ + ½	*x*_4_	−*m*
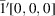	−*x*_1_	−*x*_2_	−*x*_3_	−*x*_4_ + ½	−*m*
*a*[0,0,1]	*x*_1_ + ½	*x*_2_	−*x*_3_ + ½	*x*_4_	+*m*
*m*′[0,1,0]	*x*_1_	−*x*_2_ + ½	*x*_3_	*x*_4_	−*m*
*n*[1,0,0]	−*x*_1_ + ½	*x*_2_ + ½	*x*_3_ + ½	−*x*_4_ + ½	+*m*

**Table 8:** Symmetry restrictions on the moment components and their refined values

		Symmetry restrictions			Numerical moment values (μ_B_)		
Atom	Wave	*x*	*y*	*z*	*x*	*y*	*z*
Ho1	0	*M*_*x*0_	0	*M*_*z*0_	−1.81 (8)	0	2.78 (8)
	sin1	*M*_*x*sin1_	0	*M*_*z*sin1_	−1.4 (3)	0	0.8 (2)
	cos1	*M*_*x*cos1_	0	*M*_*z*cos1_	−1.6 (2)	0	−3.73 (16)
Ho2	0	*M*_*x*0_	*M*_*y*0_	*M*_*z*0_	1.51 (7)	1.78 (4)	1.39 (7)
	sin1	*M*_*x*sin1_	*M*_*y*sin1_	*M*_*z*sin1_	−0.65 (14)	−0.83 (18)	−0.11 (15)
	cos 1	*M*_*x*cos1_	*M*_*y*cos1_	*M*_*z*cos1_	0.48 (13)	1.07 (16)	−2.02 (12)
Co	0	*M*_*x*0_	0	*M*_*z*0_	0.24 (11)	0	0.22 (7)
	sin1	*M*_*x*sin__1_	0	*M*_*z*sin__1_	−1.1 (2)	0	0.0 (2)
	cos 1	*M*_*x*cos1_	0	*M*_*z*cos1_	−1.0 (2)	0	−1.02 (19)

## Footnotes

1 The terms ‘magnetic moment’ and ‘spin’ will be used throughout the manuscript as synonyms, although in the strict sense these terms are different.
